# Quantitative metric profiles capture three-dimensional temporospatial architecture to discriminate cellular functional states

**DOI:** 10.1186/1471-2342-11-11

**Published:** 2011-05-20

**Authors:** Lindsey McKeen-Polizzotti, Kira M Henderson, Basak Oztan, C Cagatay Bilgin, Bülent Yener, George E Plopper

**Affiliations:** 1 Department of Biology, Center for Biotechnology and Interdisciplinary Studies, Rensselaer Polytechnic Institute, Troy, New York, USA; 2 Department of Computer Science, Rensselaer Polytechnic Institute, Troy, New York, USA

## Abstract

**Background:**

Computational analysis of tissue structure reveals sub-visual differences in tissue functional states by extracting quantitative signature features that establish a diagnostic profile. Incomplete and/or inaccurate profiles contribute to misdiagnosis.

**Methods:**

In order to create more complete tissue structure profiles, we adapted our cell-graph method for extracting quantitative features from histopathology images to now capture temporospatial traits of three-dimensional collagen hydrogel cell cultures. Cell-graphs were proposed to characterize the spatial organization between the cells in tissues by exploiting graph theory wherein the nuclei of the cells constitute the *nodes *and the approximate adjacency of cells are represented with *edges*. We chose 11 different cell types representing non-tumorigenic, pre-cancerous, and malignant states from multiple tissue origins.

**Results:**

We built cell-graphs from the cellular hydrogel images and computed a large set of features describing the structural characteristics captured by the graphs over time. Using three-mode tensor analysis, we identified the five most significant features (metrics) that capture the compactness, clustering, and spatial uniformity of the 3D architectural changes for each cell type throughout the time course. Importantly, four of these metrics are also the discriminative features for our histopathology data from our previous studies.

**Conclusions:**

Together, these descriptive metrics provide rigorous quantitative representations of image information that other image analysis methods do not. Examining the changes in these five metrics allowed us to easily discriminate between all 11 cell types, whereas differences from visual examination of the images are not as apparent. These results demonstrate that application of the cell-graph technique to 3D image data yields discriminative metrics that have the potential to improve the accuracy of image-based tissue profiles, and thus improve the detection and diagnosis of disease.

## Background

Errors in the structural organization and function of tissues are a major cause of many devastating human diseases, including cancer. Currently, clinicians use diagnostic profiles to distinguish between varying degrees of tissue health and disease. These profiles typically contain a combination of quantitative (e.g., expression of molecular markers, epidemiology) and qualitative (e.g., image-based assessment) data. The primary means of diagnosing most cancers is histopathological examination of a biopsy, and the resulting diagnostic profile serves as the "gold standard" in almost all cases. This examination focuses on the following traits [1]:

1. *Nuclear atypia*: The morphological atypicality of a cell (such as polymorphism, multinucleated cells, and gigantic cells) often but not always implies cancer.

2. *Cytoplasmic changes*: Higher values of the ratio of the surface area of the nucleus to that of the cytoplasm may imply cancer.

3. presence of other changes such as increased vascularity and necrosis.

4. *Cellularity*: An increase in the number/density of cells within a tissue may indicate proliferation of a cancer, or simply an increase in inflammatory processes.

5. *Cell distribution*: The location and organization of cells relative to each other is used to identify cancer. For example, cancerous brain tissues have more randomly distributed cells, whereas areas of inflammation have more evenly distributed cells. The diagnostic profile for prostate cancer includes digital rectal examination, expression levels of prostate serum antigen (PSA), and numerous image-based approaches (e.g., magnetic resonance imaging, ultrasound, CT scan, conventional biopsy/Gleason score) (reviewed in [2]). Both the incidence and mortality of prostate cancer have declined in the US and UK since the addition of PSA levels to this profile, yet the diagnostic value of the PSA test is still debated [3]. Many other molecular markers for prostate cancer are now appearing in the literature [4], though the functional roles of many are unknown. A similar situation exists for diagnosing breast cancers, such that the rate of misdiagnosis varies widely between clinicians and is nearly 40% in some cases [5].

Much of the classification errors in diagnosing solid tumors stems from incomplete tumor profiles, i.e. understanding the relationship between the functional state of a tissue and its structural organization. For example, the imaging methods used to grade the severity of solid tumors rely largely on the observations of the pathologist and qualitative metrics such as the thickness of an epithelial cell layer, atypical cell morphology, and relative uptake of contrast agents [6]. Even expression of most molecular markers is measured qualitatively, e.g., by the degree of staining with an antibody [7]. While these methods can enrich diagnostic profiles, they largely fail to address the underlying structural malfunctions that form the basis for the disease. Development of quantitative tools for image analysis and predictive modeling is thus a rapidly expanding field, showing great promise for improving diagnostic accuracy [6,8,9].

We recently developed a graph theoretical-based method, called cell-graphs, for capturing structural characteristics of histopathological images that enabled distinguishing healthy, damaged, and cancerous states of brain, breast, and bone tissues [10-12]. Our earlier studies relied on modeling functional state via the spatial organization of cell nuclei within standard histological biopsy images, and achieved accuracy equivalent to current diagnostic standards. For example, despite the visual resemblance between damaged and diseased brain tissues (both display a high cell density), the features extracted from the cell-graphs were able to distinguish between them with greater than 95% accuracy[10].

Cell-graphs are generalizations of Delaunay Triangulations that were previously used to model the spatial distribution of cells in a tissue by encoding a pair-wise relationship between two vertices [13]. In a cell-graph, nodes (or vertices) represent the cell nuclei and pairs of nodes are connected by a link (or edge) based the chemical, physical or spatial, biological relationship between them. Distance-based construction of edges was most commonly used in previous studies [10-12,14-17]. Application of graph theory to these cell-graphs provides a rich set of computational metrics that represent the structural characteristics of the underlying tissue samples. Utilization of machine learning techniques then allows us to classify different functional states of tissues. We elected to use graph theory-based methods because they have an impressive record of modeling complex relationships in numerous contexts. Real-world graphs of varying types and scales have been extensively investigated [18] in technological [19-21], social [22-28] and biological systems [29-31]. In spite of their different domains, such self-organizing structures unexpectedly exhibit common classes of descriptive spatial (topological) features [17,18,21,23,32]. These features are quantified by definition of computable metrics.

The major novelties of this study include: 1) cell-graph analysis of three-dimensional tempero-spatial tissue samples with various origins and functional states, 2) differentiation between the tissue samples based on unique structural formations relative to functional state, 3) exploitation of multi-way analysis to identify the most influential signatures that capture most of the variation in the data, and 4) establishing a correspondence between cell-graph features for *in-vitro *and *in-vivo *histology samples. The previous cell-graph work was confined to two-dimensional histology samples stained with haematoxylin and eosin. In this study we expanded our analysis to temporal analysis of 3D hydrogel models of the three most common types of tissues that develop solid tumors (epithelial, connective, and neural), to explore additional temporospatial information currently inaccessible in conventional histology samples. 2D and 3D cell culture models form the foundation for virtually all drug screening regimens and remain valid in vitro representations of human tissues[33]. Furthermore, 3D cell culture is widely used in the fields of biology and medicine to study the organization of cells in native extracellular matrix (ECM) constructs [34-36]. Likewise, cell lines with varying molecular mechanisms and protein characteristics are often used to represent a range of functional health states. Although there are limitations to *in vitro *studies, the cell lines used in this study represent a range of tissue types allowing us to directly compare the structural profiles of various functional states through analysis of cell-graph metrics. The resulting sets of cell-graph metrics that evolved over time yielded a distinct profile for each cell/tissue type, and thus have potential to identify structure-function relationship changes in a three-dimensional cell culture system. The long term goal of this study is to further understand cancer models by interpreting changes in metrics in terms of underlying changes in molecular mechanisms of cancer progression. To uncover these mechanisms, it is necessary to simplify the model in order to isolate specific cell-collagen-I interactions.

## Methods

### Cell Culture Techniques

The different cell types and their respective culture conditions are listed in Table 1. The functional categories of each cell type are listed in Table 2.

**Table 1:** Cell Culture Conditions

Name	Cell Type	Media
MCF10A	Precancerous Human	Dulbecco's Minimum Essential Media (DMEM)/F12,
	Breast Epithelial	5%Horse Serum (HS), 1% Penicillin Streptomycin (PS), 20 ng/ml Epidermal Growth Factor (EGF), .05 μg/ml Hydrocortisone, 10 μg/ml Insulin-bovine, 100 ng/ml Cholera Toxin
AU565	Human Breast Cancer HER2+/ER-	Roswell Park Memorial Institute-1640 Medium (RPMI), 10%Fetal Bovine Serum (FBS), 1%Ps
MCF7	Human Breast Cancer HER2-/ER+	Minimum Essential Media (MEM)α, 10%FBS, 1%PS, 0.01 mg/ml Insulin- bovine
MDA- MB231	Human Breast Cancer HER2+/ER2+	DMEM, 10%FBS, 1% PS
hDFB	Human Dermal Fibroblasts	DMEM, 10%FBS, 1%PS
NHA	Normal Human Astrocytes	NHA media from Lonza
U118MG	Human Glioblastoma	DMEM, 10% FBS, 1%PS
NHOst	Normal Human Osteoblast	NHOst media from Lonza
MG63	Human Osteosarcoma	DMEM, 10%FBS, 1%PS
RWPE-1	Non-tumorigenic Human Prostate	Keratinocyte serum free media from Gibco
DU145	Human Prostate Carcinoma	DMEM, 10%FBS, 1%PS

The eleven human cell lines and the corresponding culture media used in our experiments are listed in Table 1. These cells were chosen to represent connective, epithelial and neural tissue shown in Table 2. All cells were grown in conventional 2D cell culture flasks at 37°C in a humidified incubator containing 5% CO_2_. Upon reaching 80% confluency, cells were collected by treatment with trypsin-EDTA (SAFC Biosciences), washed in phosphate buffered saline (PBS), counted with a hemocytometer, and suspended in 1% collagen solution (1 × 10^6 ^cells/ml) to form hydrogels as described previously [14]. Gels were maintained under the same conditions as the 2D cultures.

**Table 2:** Cell Line Categories

	Connective	Epithelial	Neural
**Non-tumorigenic**	NHOst, hDFB	MCF10A, RWPE-1	NHA
**Cancer**	MG63	AU565, MDA-MB-231, MCF7, DU145	U118MG

### Flourescence Imaging

Gels were fixed using 3% paraformaldehyde at 6 different time points (hours): 10, 16, 24, 72, 120, 168. Each was washed with PBS, then stained with nucleic acid dye (sytox green). Images of cells encapsulated within collagen-I hydrogels were captured using a Zeiss LSM 510 META confocal microscope with a 10X dry objective. Representative Z-stack images of 100 μm thickness with 900 μm × 900 μm cross-section area were collected for five samples of each time point.

### Segmentation of Nuclei

To segment the cell nuclei, we first binarize the images. Binarization separates the image values into foreground and background classes. In our context, the foreground class represents the cell nuclei, whereas the background class represents the combination of cells and extracellular proteins. Binarization is accomplished by comparing the image values against a threshold function. Considering the large number of images that need to be processed, we employ Otsu's simple but effective automatic threshold selection algorithm[37] that determines a global (single) threshold for the image based on the histogram of image values. Each connected component in the resulting binary image corresponds to a nucleus and the coordinates of the centroids of these nuclei are calculated to identify the coordinates of the node (vertex) set for cell-graph generation.

### Generation of Cell-Graphs

After obtaining the set of vertices in the images, we construct the cell-graphs based on the pairwise nuclei distances [10-12,14-17]. We assume that a biological relation exist between two nuclei, i.e. a link (or edge) between two nodes is established, if the Euclidean distance between the corresponding centroids are less than a threshold *D*. We tested 3 thresholds: *D *= 60, 75, and 90 μm. The graphs corresponding to 60 and 90 μm turned out to be too sparse and dense, respectively. Therefore, we decided to use 75 μm as the threshold.

**Figure 1:**
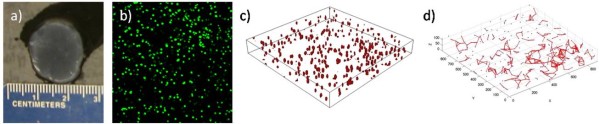
**Cell-graphs uncover hidden tissue architecture generated from 3D *in vitro *collagen-I hydrogels**. 1a shows a macroscopic image of an MG63 collagen I hydrogel following fixation. 1b displays a two-dimensional slice from 3D confocal image of hydrogel (green = nuclei). 1c is a computer generated representation of confocal image after application nuclei segmentation algorithm to identify cell location in 3D space. 1d shows how cell-graphs are built by applying graph theory to computer-generated confocal image representation.

Figure 1 illustrates the steps involved in extracting cell-graph features in 3D. Figure 1a shows an example of MG63 osteosarcoma cells, one of the eleven different types of cells representing various tissue functional states (listed in table 1), encapsulated in a collagen-I hydrogel at time 0. Figure 1b shows a two-dimensional slice of stained nuclei in a confocal fluorescence Z-stack from the hydrogel in Figure 1a. The nuclei were then identified with the application segmentation algorithm described earlier (Figure 1c) to establish nodes within the graph. We applied our cell-graph algorithm to define edges between nodes (Figure 1d) within a distance-based threshold of 75 μm resulting in a 3D cell-graph.

### Calculating Features from Cell-Graph Metrics

On each cell-graph, *G_i _(V_i _(t)*,*E_i_(t))*,where *V_i_(t) *and *E_i_(t) *represents the list of vertices and nodes at time point *t *and *i *represents the index for the cell line, we calculated 20 metrics as listed in Table 3 based on the structural features of the graphs. We then conducted an analysis in the following section to determine the metrics that have the most discriminative power between the different tissue types over time.

**Table 3:** Cell-graph metrics, interpretations, and categories.

Index	Metric Label	Metric Interpretation	Metric Category
1	Average Degree	Number of edges per node	Compactness
2	Clustering Coefficient C	Ratio of total number of edges among the neighbours of the node to the total number of edges that can exist among the neighbours of the node per node	Clustering
3	Clustering Coefficient D	Ratio of total number of edges among the neighbours of the node and the node itself to the total number of edges that can exist among the neighbours of the node and the node itself per node	Clustering
4	Clustering Coefficient E	Ratio of total number of edges among the neighbours of the node to the total number of edges that can exist among the neighbours of the node per node excluding the isolated nodes	Clustering
5	Average Eccentricity	Average of node eccentricities where the eccentricity of a node is the maximum shortest path length from the node to any other node in the graph	Compactness
6	Diameter	Maximum of node eccentricities	Compactness
7	Radius	Minimum of node eccentricities	Compactness
8	Average Path Length	Average distance between the nodes of a graph, where the distance between two nodes is the number of edges in the shortest path that connects them	Compactness
9	Hop Plot Exponent	Slope of the line fitted to the hop plot values in log-log domain, where the hop plot value for hop *h *is the number of node pairs for which the path length between the pairs is less than or equal to *h*	Compactness
10	Giant Connected Component Ratio	Ratio between the number of nodes in the largest connected component in the graph and total the number of nodes	Clustering
11	Number of Connected Components	Number of clusters in the graph excluding the isolated nodes	Clustering
12	Average Connected Component Size	Number of nodes per connected component	Clustering
13	Percentage of Isolated Points	Percentage of the isolated nodes in the graph, where an isolated node has a degree of 0	Compactness
14	Percentage of End Points	Percentage of the isolated nodes in the graph, where an isolated node has a degree of 1	Compactness
15	Number of Central Points	Number of nodes within the graph whose eccentricity is equal to the graph radius	Compactness
16	Percentage of Central Points	Percentage of the central points in the graph	Compactness
17	Average of Edge Lengths		
18	Standard Deviation of Edge Lengths		
19	Skewness of Edge Lengths	Statistics of the edge length distribution in the graph	Spatial Uniformity
20	Kurtosis of Edge Lengths		

### Three-Way Data Modeling and Analysis of Feature-Time-Cell line Joint Relationships

The data is organized to a third-order tensor with features, time, and cell-line modes whose dimensions are *I*, *J*, and *K*, respectively. An entry T*_ijk_*in the cube corresponds to the value of metric *i *at time point *j *for cell-line *k *where *i *= 1,...,20; *j *= 1,..., 6; and *k *= 1, ..., 11. Two common models in multi-way data analysis are Tucker3[38-40] and Parallel Factor Analysis (PARAFAC) [41]. A three-way tensor T ∈ ℝ^*I*×*J*×*K*^, where ℝ denotes the set of reel numbers, is decomposed using a *(P*,*Q*,*R)*-component Tucker3 model [42] as

where *P*, *Q*, and R indicate the number of components extracted from the first, second and third modes (*P *≤ *I*, *Q *≤ *J*, and *R *≤ *K*, respectively, A∈ℝ^*I*×*P*^, B∈ℝ^*J*×*Q*^, and C∈ℝ^*K*×*R*^, and are the component matrices, G∈ℝ^*P*×*Q*×*R *^is the core tensor, and E∈ℝ^*I*×*J*×*K *^represents the error term.

Parallel Factor Analysis (PARAFAC) [41] or Canonical Decomposition (CANDECOMP)[43] represents a tensor by the linear combination of rank-one tensors. An *R*-component PARAFAC model on a third-order tensor T∈ℝ^*I*×*J*×*K *^is given by

where a*_r_*, b*_r_*, and c*_r _*are the *r^th ^*columns of the component matrices A∈ℝ^*I*×*R*^, B∈ℝ^*J*×*R*^, and C∈ℝ^*K*×*R*^, respectively, E∈ℝ^*I*×*J*×*K *^is the error term, and ◦ denotes the vector outer product.

**Figure 2:**
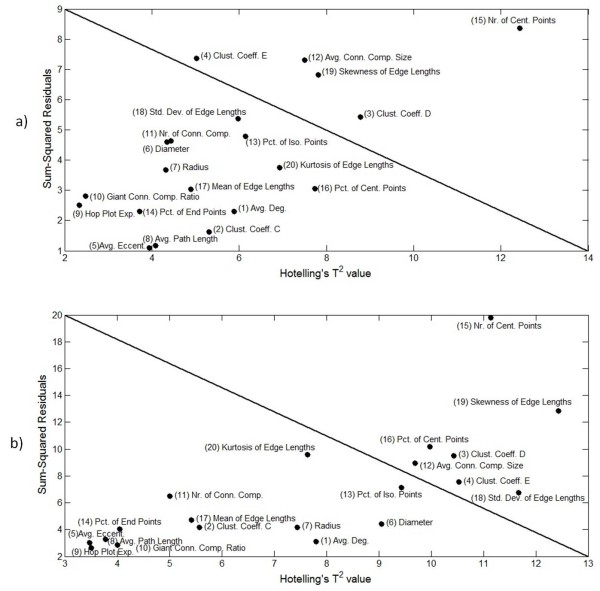
**Influence of cell-graph metrics to explain the variation in data according to the Hotelling's T^2 ^values and sum of squared residuals of each metric**. 2a and 2b show the Hotelling's T^2 ^values versus the sum of squared residuals of each metric in Tucker3 and PARAFAC model fitted data, respectively. Note that the highly influential metrics appear in the upper triangular portion of the plot.

**Figure 3:**
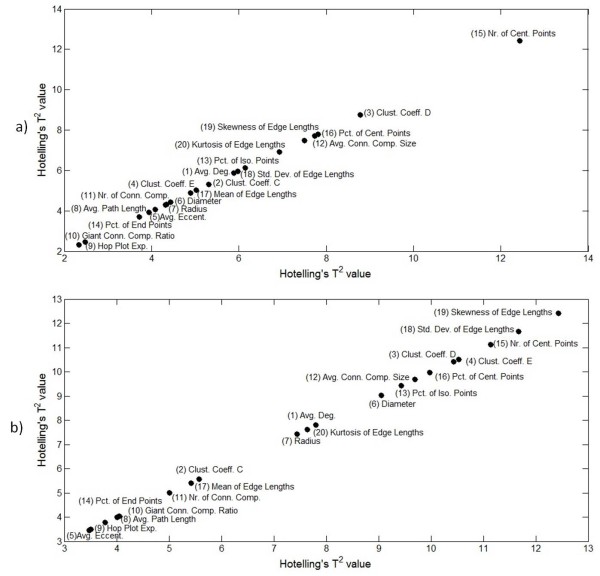
**Influence of cell-graph metrics to explain the variation in data according to the Hotelling's T^2 ^values of each metric**. 3a and 3b show the Hotelling's T^2 ^values of each metric in Tucker3 and PARAFAC model fitted data, respectively. This figure displays the metrics with increasing importance from lower left to upper right corner to discriminate between *in vitro *samples.

Prior to the model fitting, the tensor is normalized by first *centering across *the time and cell-line modes and then *scaling within *the features mode by the standard deviations[44]. In order to capture most of the variation in data, we first unfolded the tensors in each mode and determined the number of principal components that explains at least 95% of the variation in the data. The Tucker3 model was fit with 6 × 5 × 8 core tensor and the PARAFAC model was fit using 8-components to the normalized tensor where 93.7% and 89.6% of the variations in the data are captured, respectively. The analysis then focused on the feature mode in order to identify a subset of the cell-graph metrics that are more influential than the others to explain the variation in the three-way data. For this purpose, we used the Hotelling's T^2 ^statistics and the sum of squared residuals of each mode. The larger the value of these statistics, the easier it is to distinguish between the different metrics and, therefore, they are useful indicators of the influence of metrics as outliers to explain the variation in the data. These statistics are built in the MATLAB PLS Toolbox 4.0 and MATLAB Tensor Toolbox 2.4 [45]. Figure 2 shows the Hotelling's T^2 ^values versus the sum of squared residuals and Figure 3 shows only the Hotelling's T^2 ^values of each metric. From these figures, the most influential metrics are chosen as number of central points, clustering coefficient D, percentage of isolated points, standard deviation of edge lengths, and number of connected components.

### Two-Way Data Modeling and Analysis of Feature-Tissue Joint Relationships

Our histology data set contains 329 malignant and 210 benign brain samples, 128 malignant and 195 benign breast samples, and 49 malignant and 20 benign bone samples. The average of the 20 cell-graph metrics over the samples of each tumor-type is taken to construct 6 × 20 two-way data matrix. In order to determine the influence of a metric to describe the variations in the data, a singular value decomposition (SVD) -based technique is employed. First, the data is normalized by centering across the tumor-type and scaling within the features mode. Next, the data is decomposed into its *factor scores *and *loadings *using SVD. Finally, the influence of a metric is measured by the sum of absolute factor scores corresponding to the first *K *factor loadings where *K *is the number of principal components that explains at least 95% of the variation in the data. *K *is determined to be three, reflecting the number of different tissue types.

## Results

We extended our previously published cell-graph method of feature extraction into three dimensional collagen-I hydrogel cultures that remodel over time. We extracted the set of 20 quantitative features (table 3) from the generated cell-graphs. We then applied tensor analysis to the extracted features using Tucker3 and PARAFAC models to identify the features that contribute the most to discriminating between different cell/tissue temporospatial architectures over time. Figure 2a and 2b show the influence of each metric according to Hotelling's T^2 ^and sum of squared residuals scores for Tucker3 and PARAFAC models, respectively. The most important metrics are located in the upper right triangular region of these figures. Figure 3a and 3b shows Hotelling's T^2 ^scores only for Tucker3 and PARAFAC models, respectively. The metrics are displayed in increasing importance from the lower left corner to the upper right corner in this figure. We determined the five most important metrics for distinguishing between different hydrogel architectures over time based on their nuclear organization using the combination of results from Figures 2 and 3: number of central points, percentage of isolated points, number of connected components, clustering coefficient D, and standard deviation of edge lengths.

**Figure 4:**
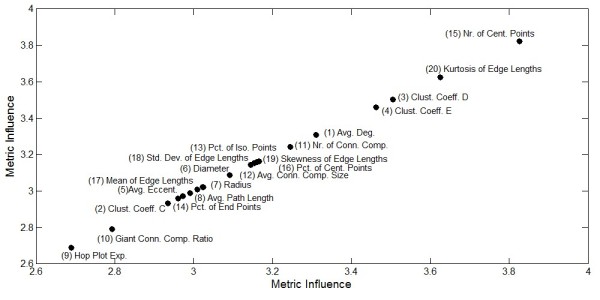
**Influence of the cell-graph metrics to describe the variations in the histology data**. This figure displays the metrics with increasing importance from lower left to upper right corner to discriminate between histology samples.

To validate our findings, we used the cell-graphs for the histology data that we analyzed using a similar framework in our earlier studies [10-12]. The discriminatory power of the extracted cell-graph metrics was successfully shown for the malignant and benign histology samples of brain [10], breast [11], and bone [12] tissues. Since these samples were surgically removed histopathology samples no temporal information is available. Thus, our histology data has two modes: tissue samples and features extracted on these samples. These data sets are obtained from 2D imaging of tissue samples from pathology department archives thus they do not have the depth information. Figure 4 shows the influence of cell-graph metrics to describe the variations in the histology data.

**Figure 5:**
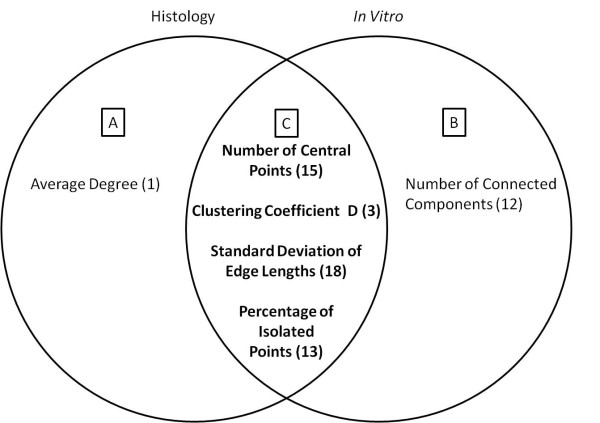
**Histology and *In Vitro *tissue both have similar as well as unique metrics that can be used to distinguish between tissue types**. The Venn diagram displays the most important metrics found by singular value decomposition and tensor analysis for the histology tissue and *in vitro *tissue images, respectively. The most discriminative metrics from the histology samples, *in vitro *samples and shared discriminative metrics are shown in figures 5a, 5b and 5c respectively. Numbers refer to feature numbers in Table 3.

Figure 5 shows the Venn diagram of the five most significant metrics for the histology and the *in-vitro *data. We found considerable overlap between the two sets of discriminative metrics as displayed by the Venn diagram in Figure 5c. This confirms that our 3D hydrogels maintain important structural properties found in histological samples.

**Figure 6:**
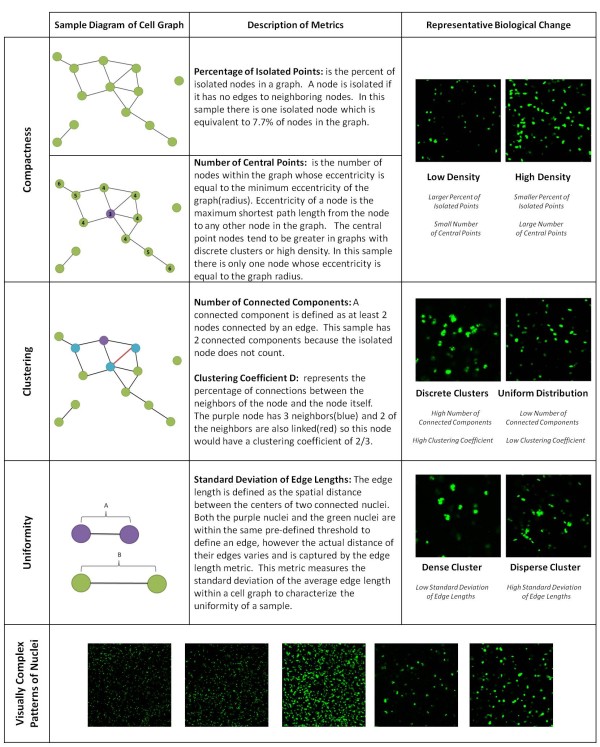
**The most significant metrics determined from the normalized tensor analysis describe the compactness, clustering and uniformity properties of tissue structure**. The diagrams on the left illustrate the metrics described in the central column. Representative images in the right column show variation for the corresponding metrics from the left column. The final row gives examples of the images analyzed in this study to show how it is difficult to quantify the important metrics by eye.

We grouped the metrics into subcategories that describe particular aspects of structural organization. The *percentage of isolated points *and *number of central points *reflect the overall compactness of a cell-graph, as shown in Figure 6. The compactness metrics can quantify changes in cell density over time that we represented in the biological images in the top right of Figure 6. The change in cell density from low to high results in higher compactness and is captured by an increase in number of central points and a decrease in the percent of isolated points.

The second subcategory of descriptive metrics, *number of connected components *and *clustering coefficient D*, capture the extent of cell clustering in a sample. As seen in the representative biological images in the clustering row of Figure 6, samples with discrete clusters have a high number of connected components and a high clustering coefficient. On the contrary, uniformly distributed cells (non-clustering) have a low number of connected components and a low clustering coefficient, i.e. a majority of the cells in the sample are connected within a single connected component. The *standard deviation of edge lengths *describes the consistency in the distance distribution between the nuclei, thus establishing the level of uniformity in the sample. A sample with uniform-dense clusters, as shown in the lower right of Figure 6, results in a low standard deviation edge of lengths and high uniformity. Alternatively, a sample with a disperse cell cluster distribution yields a high standard deviation edge of lengths and a lower uniformity.

The biological images from Figure 6 represent the extremes of the three subcategories of metrics, compactness, clustering and uniformity. In reality, the hydrogel architecture of different cell types typically lies between the extremes of each metric, and changes over time as the structure develops. The last row in Figure 6 gives examples of the variety of visually complex patterns of cell nuclei, from 5 different cell types, analyzed as part of this study. In these instances, visual inspection of hydrogel architecture images does not distinguish between the cell types and time points. Therefore, we used the cell-graphs and quantified the changes in metric values over time to differentiate the cell types from each other.

**Figure 7:**
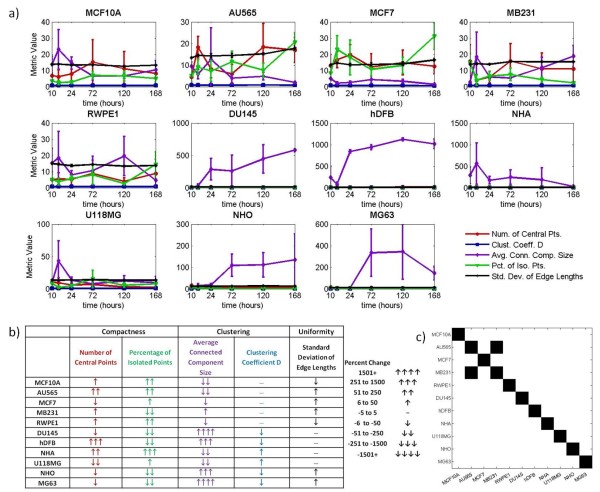
**The most significant metrics capture structural differences to generate a unique metric profile for each cell type**. 7a plots the raw data and standard deviation bars of the most important metrics from the generated cell-graphs for each cell type over time. Due to the scale of the graphs in 7a it is difficult to see small changes in metric values, however these changes are captured by the percent changes shown in 7b. 7b was generated by first calculating the averages of the data points in 7a at hour 10 and 16 for each sample as well as the averages for the data points at hours 120 and 168 (the first and last two time points in the graphs, respectively). These averages were then used to determine the percent change of each metric for each cell type over time. The key to 7b shows how arrows represent varying degrees of percent change in the table. Figure 7c shows the results of the combination of two-sample Kolmogorov-Smirnov test results for the five most significant metrics. The cell-line pairs that belong to similar probability distributions are shown with black squares. Note that the cell-lines are in exact agreement with themselves.

Figure 7 shows that the data trends from five cell-graph metrics are sufficient to distinguish between the hydrogel architectures formed by eleven different cell types. In Figure 7a, the raw data of the five metrics (determined by tensor analysis, Figures 2 and 3) were plotted for each cell type over time. Individual plots for each metric in Figure 7a can be found in Additional files 1, 2, 3, 4 and 5. To directly compare the metric trends between cell type architectures, we generated Figure 7b as a visual representation of the same data in Figure 7a. Figure 7b shows that the metrics for each cell type exhibit a distinct pattern of value changes over time. The patterns indicate both the direction of change (i.e. up arrow, down arrow or flat line) and relative magnitude (i.e. number of arrows). In addition, we performed two-sample Kolmogorov-Smirnov tests between pairs of cell-lines to investigate if the corresponding metrics belong to the same probability distribution function. For each pair of cell-lines, the test is performed over the five most significant features. If the two cell-lines come from the same probability distributions, the result of the test is 0, and 1 otherwise. The results of the five tests are combined by logical *OR *operation. Figure 7c shows the results for the 11 cell lines used in our experiment at 10% significance level. It is clearly seen that most of the cell-lines belong to different probability distributions that the influential metrics are effective in distinguishing between the different cell-types. From this large set of data, only the data from the closely related AU565 and MB231 breast cancer cells to lack statistical significance. This data is capable of discriminating all cell lines from each other except between AU565 and MB231.

The first six cell types listed in Figure 7b are of epithelial origin (breast and prostate cells) representing a range of cancer grades from pre-cancerous to metastatic. Each has a unique metric profile. The standard deviation of edge lengths metric values distinguish the MCF10A (pre-cancerous) breast epithelial cells from the AU565 breast cancer cells because they trend in opposite directions over time. Like the AU565 cells, the MCF7 cells also show similar trends to the MCF10A cells for the percentage of isolated points and average connected component size metrics. However, in addition to the opposing standard deviation of edge lengths trend that distinguishes the AU565 from the MCF10A cells, the MCF7 cells also show an opposing decreasing trend in the number of central points. The metric trends for MB231 cells also differ from those for MCF10A. While the uniformity metric trends for MB231 cells resemble those for the MCF7 cells, the metrics that capture clustering and compactness show opposite trends. Compared to the other breast cancer cells, the percentage of isolated points and average connected component size for MB231 cells show opposite trends. Interestingly, the non-tumorigenic RWPE1 prostate cells and the MCF10A breast cells have nearly identical metric trends with only a slight difference in the magnitude of the average connected component size. Likewise, the metric changes between non-tumorigenic RWPE-1 prostate cells and metastatic DU145 prostate cells are similar to those seen between the non-tumorigenic MCF10A breast cells and the breast cancer lines.

The non-tumorigenic NHA cells and the cancerous U118 cells are glial cells from brain tissue origin. The brain hydrogels exhibit a pattern of metric trends which differentiates them from the hydrogels of other cell/tissue types in this study.

Although the pattern of metric trends is similar in both of the brain hydrogels, the NHA cells are distinguishable from their cancerous counterpart (U118) due to the opposite trend in the number of central points and the magnitude change in the percentage of isolated points. Both of the metrics that distinguish between the non-tumorigenic and cancerous brain cells are measures of compactness. Similar to the brain cells, the representative bone hydrogel architecture (NHOst and MG63) have a distinct set of metric trends, which differentiate them from the other tissue types in the study. The NHOst and MG63 are distinguishable from each other due to the magnitude of the average connected component size, a measure of clustering. Interestingly, the DU145 cells (metastatic prostate epithelial) show similarity between the bone cells (NHOst and MG63) and the fibroblasts (hDFB). The only variations between the DU145 and bone cells are the trends of the standard deviation of edge lengths. The DU145 compared to the hDFB only show different trends in the number of central points. Similarly, the MB231 cell line (metastatic breast epithelial) shows the same pattern of metric trends as the hDFB (fibroblasts), with differences in magnitude of change and slight variation in the clustering coefficient D and standard deviation of edge lengths.

## Discussion

A hallmark of all complex tissues is carefully organized cell and ECM architecture. We believe this architecture is determined, at least in part, by a set of organizational "rules" that determine how cells orient with respect to each other. According to our model, both damaged and cancerous tissues exhibit architectures that deviate significantly from the non-tumorigenic state dictated by these rules, but it is very difficult to quantify changes in these rules by eye. Teasing out the characteristic differences between different functional states in a tissue thus benefits from identifying and understanding the biological foundation for these rules.

This study represents the first attempt at defining these rules, by assigning rigorous quantitative metrics to architectural properties of 3D hydrogels containing distinct cell types. 3D collagen-I hydrogels provide elements of tissue structure which are not obtainable in traditional 2D histology imaging. In this model system, cells from diverse tissue origins interact differently with the collagen-I ECM and each other, resulting in a range of tissue architectures over time. The features extracted from the cell-graphs of 3D confocal images of cell nuclei from the hydrogels are analyzed using Tucker3 model to extract signature graph features. While it is very difficult to quantify important metrics from our images by eye, our computational approach uncovers hidden relationships in these images to discriminate between cell types in 3D, over time.

Our method improves upon histopathological image analysis using nuclear distance-based cell-graphs [13] to include more aspects of tissue structure-function relationships. Comparison of the Singular Value Decomposition analysis of our 2D histology data and tensor analysis of our 3D *in vitro *feature sets revealed partial overlap of the most significant discriminating metrics. In Figure 5a, the *average degree *metric represents the connectivity and compactness of the 2D histology samples is the most significant for distinguishing between tissue types. The most significant metric in Figure 5b, *number of connected components*, characterizes the clustering of the sample. The overlapping metrics in Figure 5c show that histology and *in vitro *samples share metrics that characterize the compactness, clustering and uniformity of cellular structure organization in order to distinguish between tissue types.

With the metrics determined by tensor analysis, we were able to distinguish multiple functional states of tissues based solely on their nuclear organization in a 3D collagen-I hydrogel. Using the metric profiles for each cell type (Figure 7b), we are able to discriminate different grades of breast and prostate cancer due to a variety of characteristic differences in trends between cell types. The profiles also successfully distinguish non-tumorigenic brain and bone tissue organization from their cancerous counterparts. However, it is only a change in magnitude of the average connected component size that is able to distinguish between the non-tumorigenic and cancerous bone cells. In the future, we will seek to identify new metrics that better distinguish the differences between mesenchymal tissues.

Our findings present an intriguing possibility, that the data in this study may be capturing features of the epithelial to mesenchymal transition (EMT). EMT is defined as a cellular change from epithelial phenotype to mesenchymal phenotype, involving a loss of adherens junctions, change in intermediate filament expression, and an increase in cell mobility [46-49]. These cellular changes tend to result in a more aggressive, metastatic cancer. While EMT is a characteristic of epithelial tumor progression, it is difficult to quantify using structural changes or molecular markers[50].

In this study, we have included cell types which represent varying stages of EMT, based on their protein expression profile. The breast cancer cell types (MCF10A, AU565, MCF7, and MB231) represent progressive cancer grades from precancerous to metastatic (respectively). Interestingly, our metric profiles capture differences in metric trends between each cell type. The first change between MCF10A and AU565 represents a change in uniformity of cell distribution. The AU565 and MCF7 cell organizations differ by a change in the trend for the *number of central points *metric, representing a change in the clustering of the cells within tissue architectures. MB231 are further discriminated from the MCF7 cells by an increase in the compactness of the tissue, as demonstrated by the change in *number of central points *and *percentage of isolated points*. MB231 also shows a change in *average connected component size *trend compared to the other breast cancer lines. In addition, the MB231 metric profile shares similar trends as the mesenchymal fibroblast cell line as opposed to it's' breast cancer counterpart, MCF10A. The DU145 cells show remarkably similar metrics to both the osteogenic (NHOst, MG63) and fibroblast cells, with a change in only one metric trend between them. The resemblance in trends between the MB231 and DU145 cells with the mesenchymal tissue organizations (particularly the osteogenic lines NHOst and MG63) may reflect the frequency with which breast and prostate cancer metastasizes to bone.

## Conclusions

Collectively, our findings demonstrate that our three-dimensional cell-graph methodology is capable of discriminating between structural patterns of cellular organization in model tissues representing different grades of tumor progression and tissue origin that cannot be quantified by eye. The distinguishing features are based on three-mode tensor analysis of graph theoretical properties calculated for each cell type over time. By extending the sensitivity of image analysis and tissue modeling to uncover diagnostic, hidden, temporospatial relationships between cells in model tissues, we feel this is a significant step towards enriching diagnostic profiles for disease. Such enhanced profiles have the potential to improve diagnostic accuracy and identify hidden traits that may suggest new therapeutic interventions.

## Competing interests

The authors declare that they have no competing interests.

## Authors' contributions

LMP and KH carried out the generation and maintenance of cellular 3D collagen I hydrogel cultures, collected confocal fluorescent microscopy images, participated in the design of the study, generation of figures and tables, analysis and interpretation of data, draft, and revision of the manuscript. BO carried out the generation and analysis of 2D histology image and contributed to the revising of the manuscript. CB carried out the 3D fluorescent image segmentation, cell-graph generation, and metric extraction. BY carried out the tensor analysis and contributed to drafting the methods and revising manuscript. GP participated in the design of the study, analysis and interpretation of data, and draft of the manuscript. All authors read and approved the final manuscript.

## Pre-publication history

The pre-publication history for this paper can be accessed here:

http://www.biomedcentral.com/1471-2342/11/11/prepub

## Supplementary Material

Additional file 1 **Figure S1- Raw data plots for the number of central points metric**. Shows the raw data for the number of central points metric plotted for each cell type individually over time. Click here for file

Additional file 2 **Figure S2- Raw data plots for the clustering coefficient D metric**. Shows the raw data for the clustering coefficient D metric plotted for each cell type individually over time. Click here for file

Additional file 3 **Figure S3- Raw data plots for number the average connected component size metric**. Shows the raw data for the average connected component size metric plotted for each cell type individually over time. Click here for file

Additional file 4 **Figure S4- Raw data plots for the percentage of isolated points metric**. Shows the raw data for the percentage of isolated points metric plotted for each cell type individually over time. Click here for file

Additional file 5 **Figure S5- Raw data plots for the standard deviation of edge lengths metric**. Shows the raw data for the standard deviation of edge lengths metric plotted for each cell type individually over time. Click here for file
