# Supplementary figures and images for: Quantitative metric profiles capture three-dimensional temporospatial architecture to discriminate cellular functional states
Source: BMC Med Imaging. 2011 May 20;11:11. doi: 10.1186/1471-2342-11-11 (PMC3125246; doi:10.1186/1471-2342-11-11)

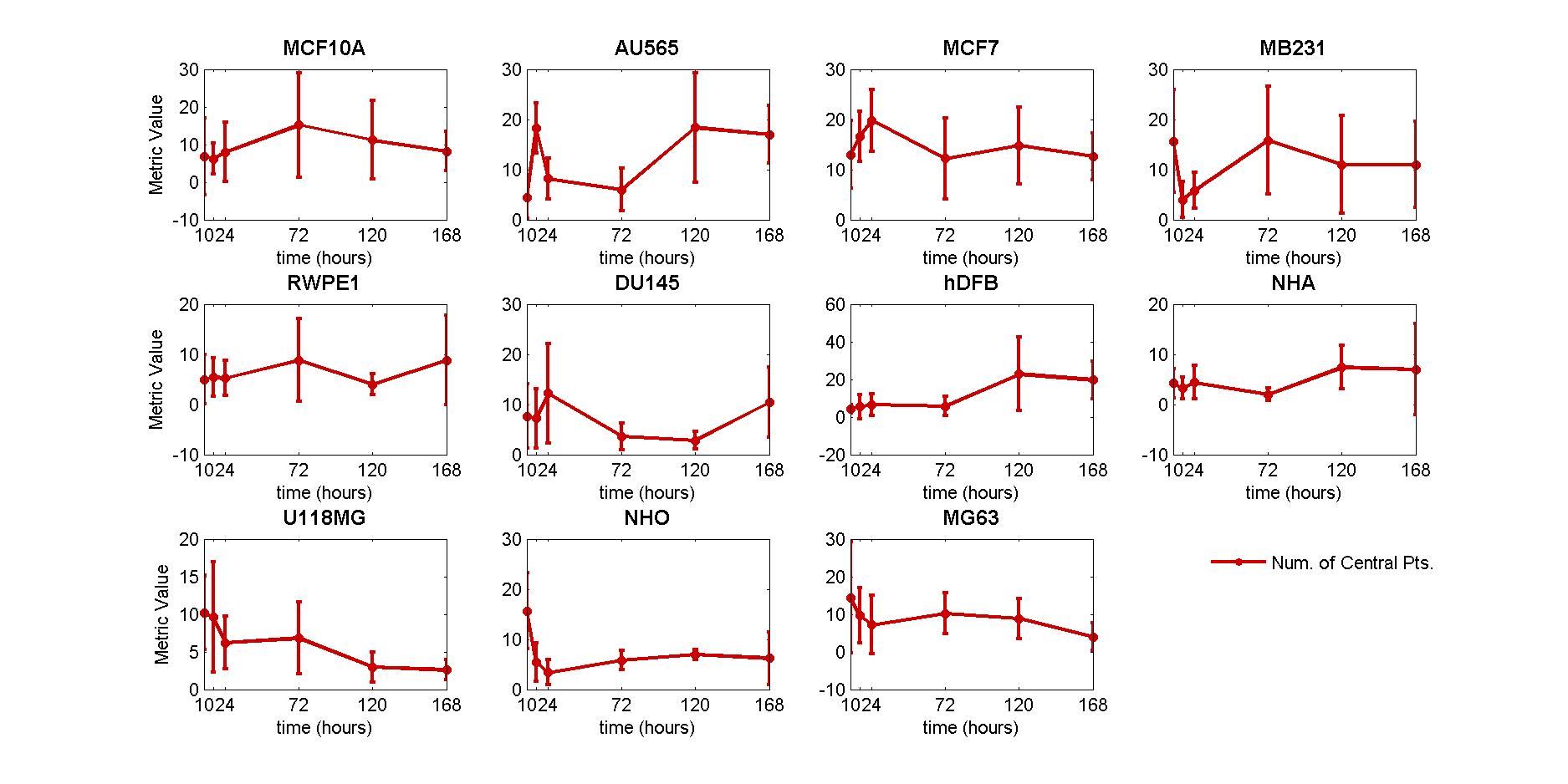

Supplement: Additional file 1 — Figure S1- Raw data plots for the number of central points metric. Shows the raw data for the number of central points metric plotted for each cell type individually over time. [file 1471-2342-11-11-S1.JPEG]

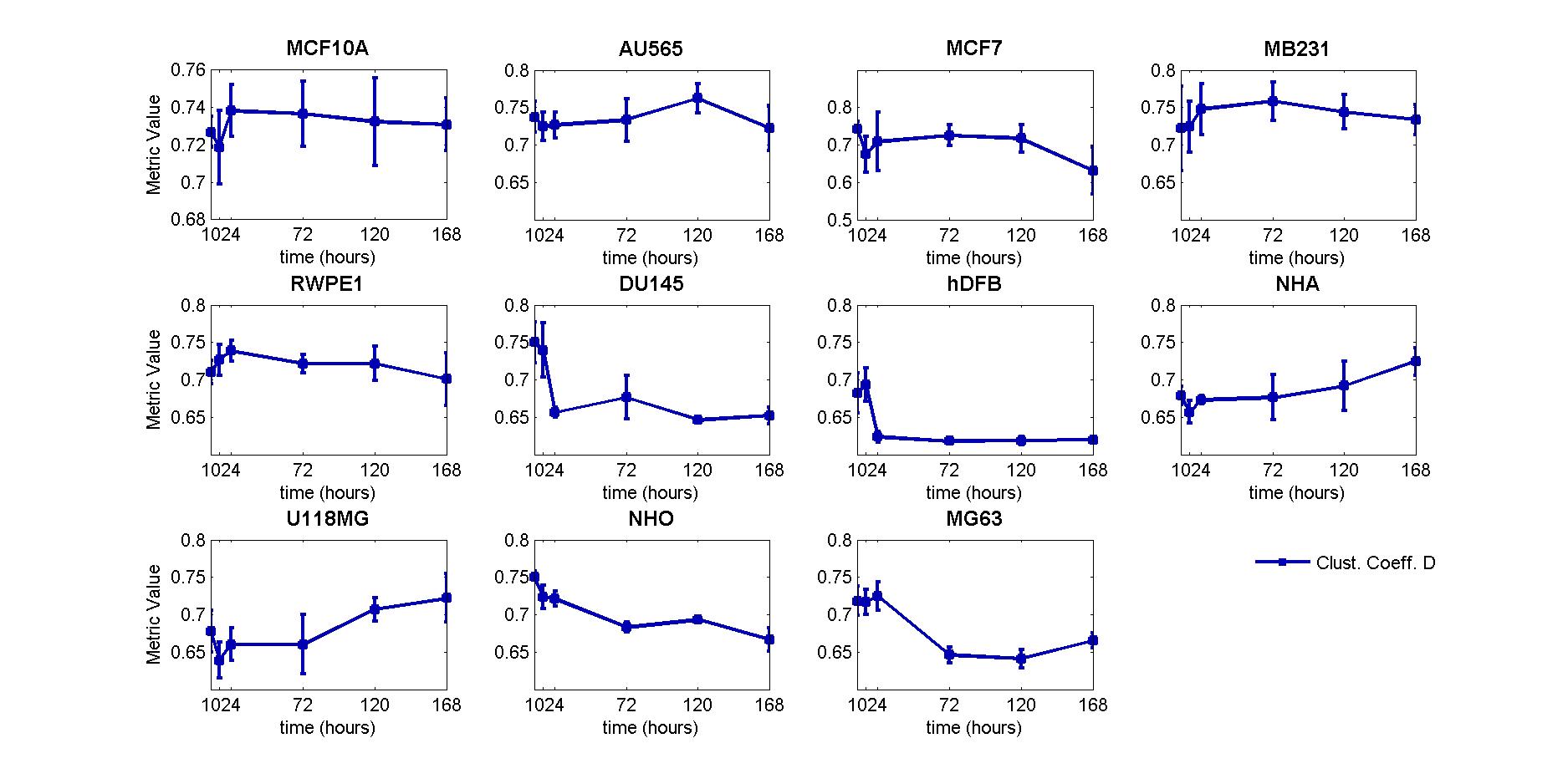

Supplement: Additional file 2 — Figure S2- Raw data plots for the clustering coefficient D metric. Shows the raw data for the clustering coefficient D metric plotted for each cell type individually over time. [file 1471-2342-11-11-S2.JPEG]

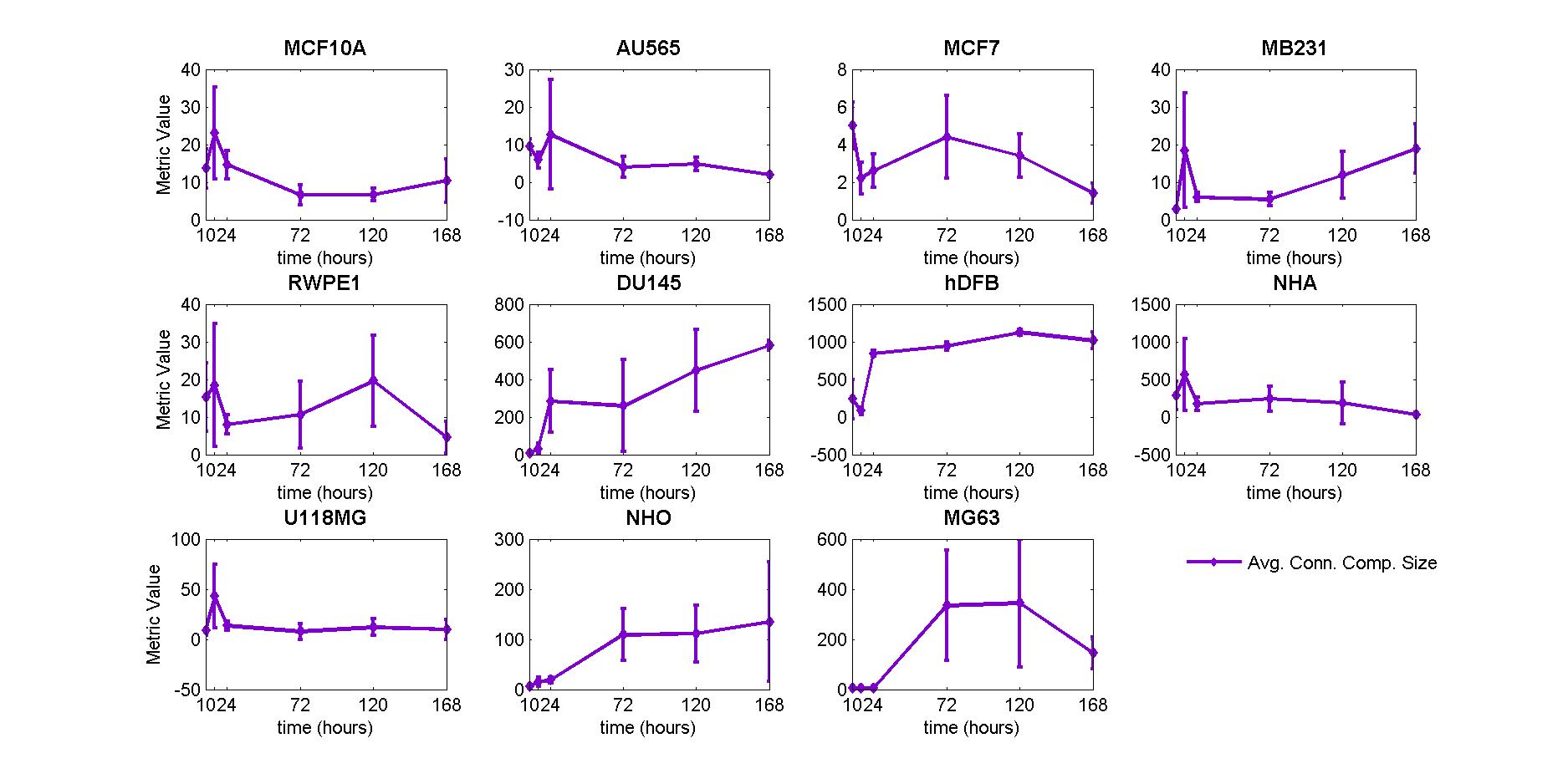

Supplement: Additional file 3 — Figure S3- Raw data plots for number the average connected component size metric. Shows the raw data for the average connected component size metric plotted for each cell type individually over time. [file 1471-2342-11-11-S3.JPEG]

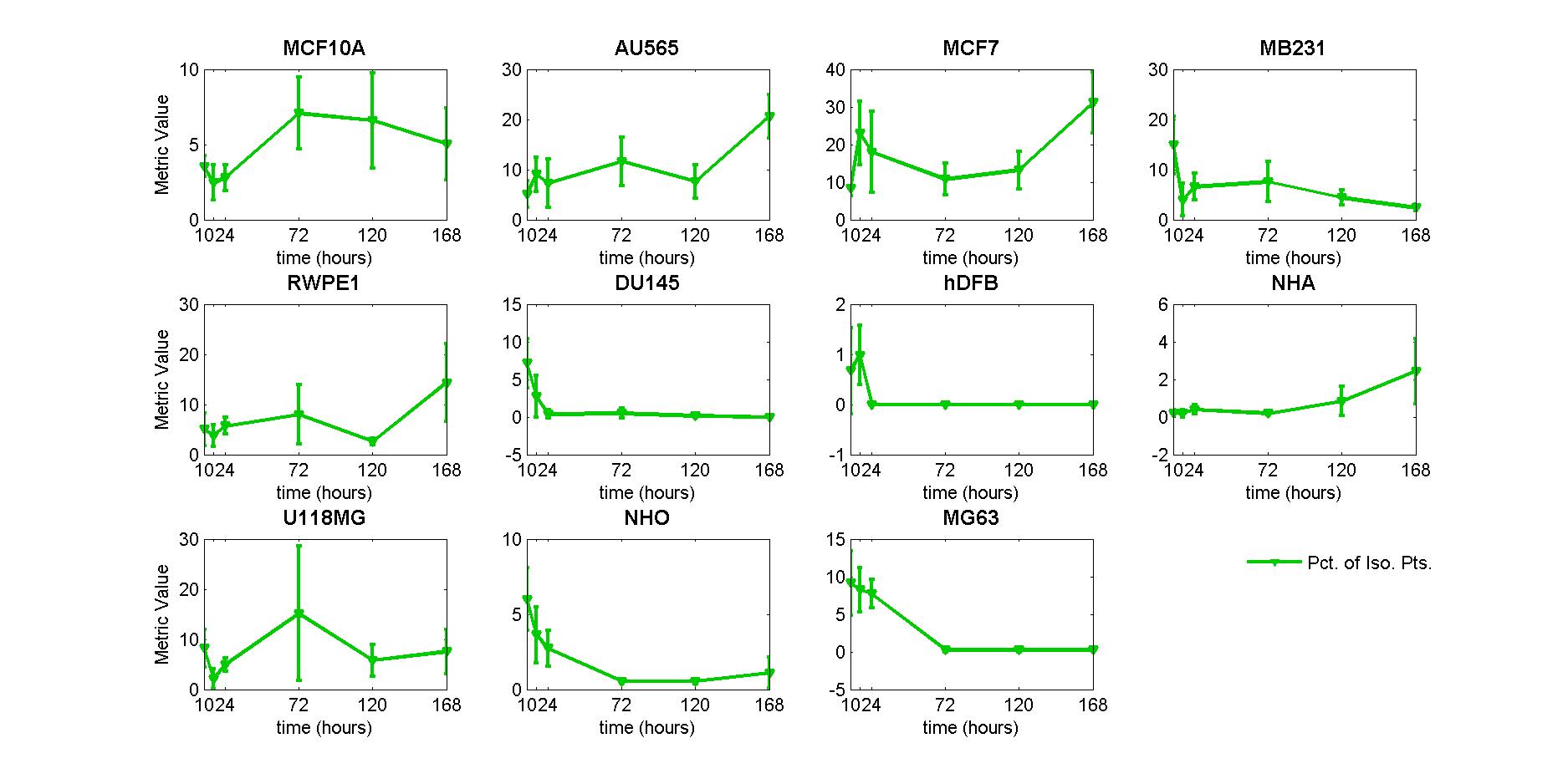

Supplement: Additional file 4 — Figure S4- Raw data plots for the percentage of isolated points metric. Shows the raw data for the percentage of isolated points metric plotted for each cell type individually over time. [file 1471-2342-11-11-S4.JPEG]

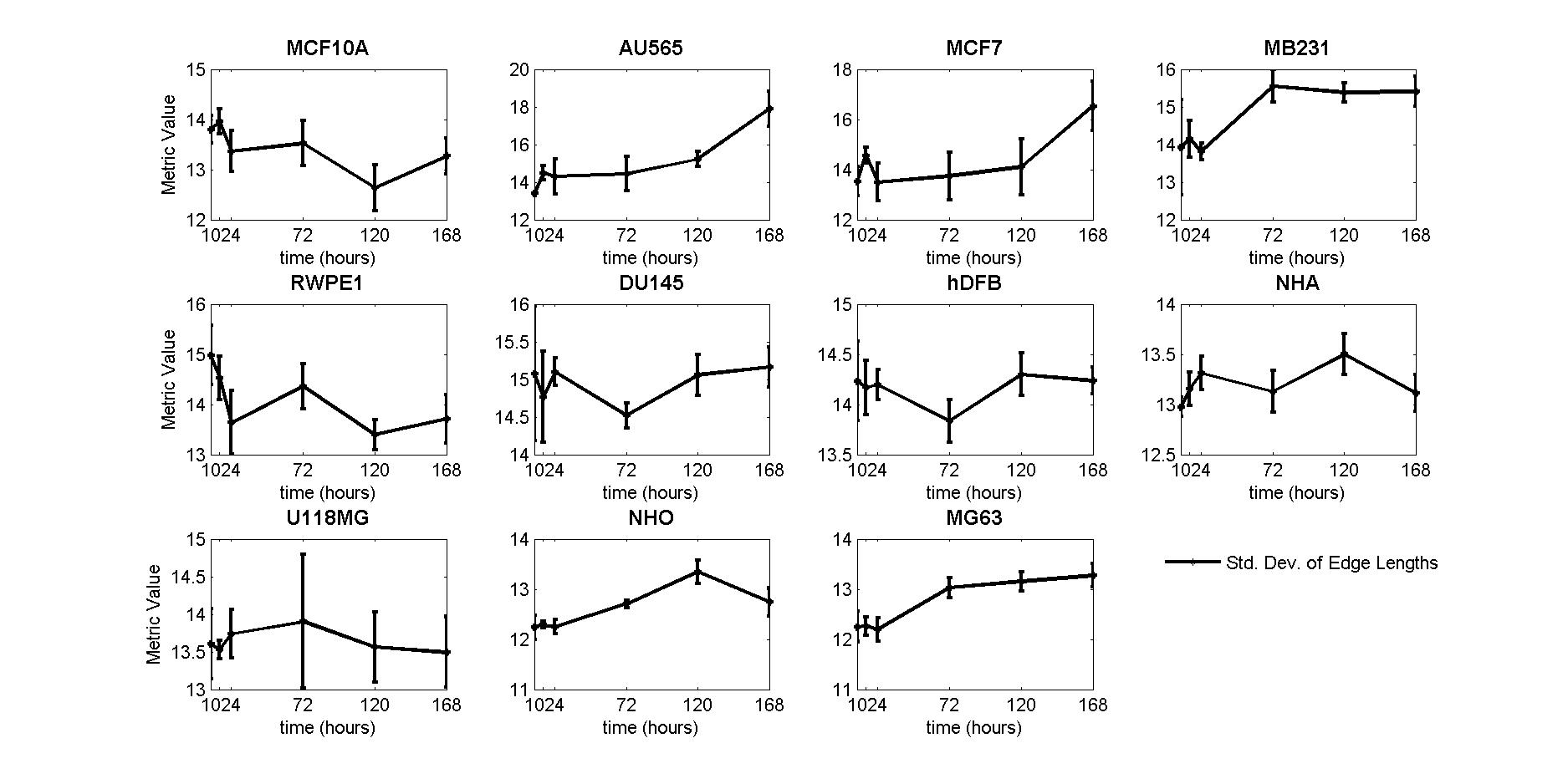

Supplement: Additional file 5 — Figure S5- Raw data plots for the standard deviation of edge lengths metric. Shows the raw data for the standard deviation of edge lengths metric plotted for each cell type individually over time. [file 1471-2342-11-11-S5.JPEG]
